# Alterations in blood glucose and plasma glucagon concentrations during deep brain stimulation in the shell region of the nucleus accumbens in rats

**DOI:** 10.3389/fnins.2013.00226

**Published:** 2013-12-10

**Authors:** Charlene Diepenbroek, Geoffrey van der Plasse, Leslie Eggels, Merel Rijnsburger, Matthijs G. P. Feenstra, Andries Kalsbeek, Damiaan Denys, Eric Fliers, Mireille J. Serlie, Susanne E. la Fleur

**Affiliations:** ^1^Department of Endocrinology and Metabolism, Academic Medical Center, University of AmsterdamAmsterdam, Netherlands; ^2^Department of Psychiatry, Brain Center Rudolf Magnus, University Medical CenterUtrecht, Netherlands; ^3^Department of Translational Neuroscience, Brain Center Rudolf Magnus, University Medical Center UtrechtUtrecht, Netherlands; ^4^Department of Psychiatry, Academic Medical Center, University of AmsterdamAmsterdam, Netherlands; ^5^Department of Neuromodulation and Behaviour, Netherlands Institute for Neuroscience, an institute of the Royal Netherlands Academy of Arts and SciencesAmsterdam, Netherlands; ^6^Department of Hypothalamic Integration Mechanisms, Netherlands Institute for Neuroscience, an institute of the Royal Netherlands Academy of Arts and SciencesAmsterdam, Netherlands

**Keywords:** deep brain stimulation (DBS), nucleus accumbens shell, lateral hypothalamic area, glucose, glucoregulatory hormones, neural activity

## Abstract

Deep brain stimulation (DBS) of the nucleus accumbens (NAc) is an effective therapy for obsessive compulsive disorder (OCD) and is currently under investigation as a treatment for eating disorders. DBS of this area is associated with altered food intake and pharmacological treatment of OCD is associated with the risk of developing type 2 diabetes. Therefore we examined if DBS of the NAc-shell (sNAc) influences glucose metabolism. Male Wistar rats were subjected to DBS, or sham stimulation, for a period of 1 h. To assess the effects of stimulation on blood glucose and glucoregulatory hormones, blood samples were drawn before, during and after stimulation. Subsequently, all animals were used for quantitative assessment of Fos immunoreactivity in the lateral hypothalamic area (LHA) using computerized image analysis. DBS of the sNAc rapidly increased plasma concentrations of glucagon and glucose while sham stimulation and DBS outside the sNAc were ineffective. In addition, the increase in glucose was dependent on DBS intensity. In contrast, the DBS-induced increase in plasma corticosterone concentrations was independent of intensity and region, indicating that the observed DBS-induced metabolic changes were not due to corticosterone release. Stimulation of the sNAc with 200 μA increased Fos immunoreactivity in the LHA compared to sham or 100 μA stimulated animals. These data show that DBS of the sNAc alters glucose metabolism in a region- and intensity- dependent manner in association with neuronal activation in the LHA. Moreover, these data illustrate the need to monitor changes in glucose metabolism during DBS-treatment of OCD patients.

## Introduction

Deep brain stimulation (DBS) of the nucleus accumbens (NAc) is used to treat obsessive compulsive disorder (OCD), depression and addiction (Denys et al., 2010; Bewernick et al., 2012; Muller et al., 2013) and is currently under investigation for the treatment of eating-disorders (eg., Halpern et al., 2008). Although the precise mechanisms through which DBS exerts its effects remain to be elucidated, recent data from human and animal studies suggest that DBS directly affects neuronal network activity (McCracken and Grace, 2007; Vandehey et al., 2010; Tan et al., 2011; Figee et al., 2013) and alters neurotransmitter release (van Dijk et al., 2012; Halpern et al., 2013).

Effective pharmacological treatment of OCD, such as anti-depressants and serotonin reuptake inhibitors target the dopaminergic- and serotoninergic system. This suggests that modulation of these neurotransmitter systems could well be involved in the effects of DBS. Unfortunately, drugs targeting these neurotransmitter systems increase the risk to develop type 2 diabetes, through direct modulation of glucose metabolism, independent of alterations in body weight. In addition, DBS of the NAc has been associated with changes in food intake (van der Plasse et al., 2012; Halpern et al., 2013). In light of these findings, and the current interest in DBS as a treatment for eating-disorders (Benabid and Torres, 2012), it is imperative to determine whether DBS might influence glucose metabolism and thus induce side effects.

Central control of glucose metabolism is mediated by multiple brain areas and neurotransmitter systems that include serotonergic neurons in the raphe nucleus, noradrenergic neurons in the locus coeruleus, and hypothalamic nuclei that project, directly and indirectly to brainstem nuclei that regulate autonomic outflow (Lechin and van der Dijs, 2006; Marino et al., 2011). Of these, hypothalamic nuclei are ideally situated to sense and integrate peripheral metabolic signals and regulate autonomic tone to maintain a positive energy balance. With respect to possible NAc DBS-induced alterations in glucose metabolism and food intake, early viral tracing experiments in rats revealed a neural connection between the NAc (part of the ventral striatum) and pancreas (Buijs et al., 2001). Specifically, the shell region of the NAc (sNAc) projects to the lateral hypothalamic area (LHA), directly as well as via the ventral pallidum (Zahm and Brog, 1992). The LHA, in turn, projects to the dorsal motor nucleus of the vagus, the ventral lateral medulla and preganglionic spinal cord neurons, all of which project to the pancreas to regulate endocrine pancreatic functions, but also project to the liver and thus may alter glucose metabolism (Buijs et al., 2001; Berthoud, 2004; Wu et al., 2004; Yi et al., 2010).

It is well documented that the projection between the sNAc and the LHA plays a role in food directed behavior (Kelley and Swanson, 1997; Stratford and Kelley, 1999). Recently, van der Plasse et al. (2012) showed that DBS of the sNAc in free-fed rats with access to normal chow elicits feeding behavior. However, to date, a role for this neuroanatomical connection in the regulation of glucose metabolism has not been investigated. We hypothesized that the neural connection between the sNAc and pancreas (and liver) is functional in glucose metabolism. To investigate this hypothesis, we implanted stimulation electrodes in the sNAc of rats and studied the effects of local stimulation on blood glucose and glucoregulatory hormones. In addition, to test if the LHA is involved in the effects of sNAc stimulation we measured the Fos response in the LHA following DBS of the sNAc, as a marker for neuronal activity. We subjected rats to 1h stimulation at two different intensities. Prior to-, during-, and after cessation of stimulation, blood samples were drawn and concentrations of blood glucose and plasma glucoregulatory hormones were measured. Subsequently, brain sections were stained and Fos activation in the LHA was quantified. This study shows for the first time the effects of electrical stimulation in the sNAc on neural activity in the LHA and on glucose metabolism.

## Methods

### Animals

Twenty five male Wistar rats (250–280 g; Harlan, Horst, the Netherlands) were individually housed in Plexiglas cages in a temperature (20 ± 2°C), humidity (60 ± 2%) and light controlled room with a 12/12 h light-dark schedule (lights on at 7:00 h AM). All animals had *ad libitum* access to laboratory chow (Teklad Global 18% Protein Rodent Diet, Harlan, Horst, Netherlands) and tap water prior to testing.

Rats were adapted to handling in the period prior to surgery. The experiment was performed in the rat's home cage. The experiment was approved by the Committee for Animal Experimentation of the Academic Medical Center of the University of Amsterdam, Netherlands.

### Surgery

Rats were anaesthetized with an i.p. injection of 80 mg/kg Ketamine (Eurovet Animal Health, Bladel, Netherlands), 8 mg/kg Rompun® (xylazine, Bayer Health Care, Mijdrecht, Netherlands) and 0.1 mg/kg Atropine (Pharmachemie B. V., Haarlem, Netherlands), after which an intra-atrial silicone catheter was implanted in the jugular vein, according to the method of Steffens (1969). After catheter implantation, rats were bilaterally implanted with bipolar electrodes (dual stainless steel electrodes, 300 μm length, 125 μm diameter, distance between poles was 100 μm, 325 μm of the end of the electrodes was stripped; PlasticOne) aimed at the sNAc (A + 1.44 mm, L + 3 mm, V −7.3 mm, angle 17°), using a stereotaxic apparatus (Kopf). Catheters and electrodes were fixed on the skull with dental cement. Rats received a recovery period of 7 days.

### Stimulation

Four hours prior to stimulation food was removed (i.e., at 8:00h AM). Animals were connected to the blood-sampling catheter and electrode implants were attached to stimulation cables which were, via an electrically-shielded dual channel swivel (Med Associates, St Albans, VT, USA), connected to stimulation equipment. The sampling catheter and cables were kept out of reach by means of a counterbalanced beam. This allowed the animals to move freely during the experiment and allowed all manipulations to be performed outside the cages without handling the animals.

On experimental days a total of 25 rats were subjected to 60 min of either 100 μA (*n* = 12) or 200 μA (*n* = 13) or sham (all animals) stimulation. Each animal served as its own control and was, controlled for body weight, randomly assigned to an experimental group. Each experimental day all three stimulation conditions were applied. Rats received 7 days of recovery before being switched in experimental condition.

Stimulations were performed with a digital stimulator (DS8000, World Precision Instruments, Sarasota, USA) and stimulus isolator (DLS100, World Precision Instruments, Sarasota, USA). Stimulation parameters were as follows; biphasic square pulses, 60 μ s duration, 200 μ s ’zero' time, frequency 130 Hz. Blood samples were drawn prior (*t* = −1 min, baseline) during (*t* = 5, *t* = 10, *t* = 15, *t* = 30, *t* = 60 min) and following cessation of stimulation (*t* = 90 and *t* = 120 min).

### Analytical methods

Blood glucose concentrations were measured directly during the experiment, using a custom glucose meter (Freestyle Freedom Lite, Abbot, Hoofddorp, Netherlands). Blood samples were immediately chilled on ice in Eppendorf tubes with 5 μ L heparin: saline (10x) solution and centrifuged at 4°C (15 min, 3000 rpm). Plasma samples were stored at −20°C until further analysis. Plasma insulin, glucagon and corticosterone concentrations were measured using radioimmunoassay kits (Millipore, St Charles, MO, USA and Biochemicals, Costa Mesa, CA, respectively). The amount of sample-, standards-, label-, antibody and precipitating reagent, described in the manufacture's protocol, were divided by four. The variation-coefficient of the immunoassays was < 10%.

### Histology and immunocytochemistry

At the end of the experiment (*t* = 120), animals were anaesthetized with a CO_2_/O_2_ mixture (6:4) followed by 100% CO_2_ and killed by decapitation. Brains were then rapidly removed, frozen on dry ice and stored at −80^°^. Brain tissue was cut on a cryostat in 35 μm sections. Sections were collected on gelatin coated slides and fixed for 10 min in 4% paraformaldehyde at room temperature. For verification of electrode placement, slides were Nissl-stained after fixation and examined with a microscope to determine precise location of the electrodes. Given the functional specificity of the sNAc in the (para) sympathetic projection to the pancreas and liver, electrode placement was considered misplaced when electrode tips were observed outside the sNAc according to the delineation of Paxinos and Watson (1998).

For immunohistochemical staining, sections were incubated with 10% methanol, 3% H_2_O_2_ in Tris-buffered saline (TBS, 0.06 M Tris, 0.2 M NaCl, pH 7.6) for 10 min. Slides were then rinsed in TBS (3 times, 10 min) and incubated overnight at 4°C with goat anti-Fos IgG (1:1500; Santa Cruz Biotechnology, Inc., California) diluted in supermix (SUMI, 0.25% gelatin, 0.5% Triton X-100 in TBS (pH 7.6)). Following incubation, slides were rinsed in TBS (3 times, 10 min), incubated for 1 h in biotinylated horse anti-goat IgG (1:400 in SUMI; Vector Laboratories Inc., Burlingame, CA), rinsed in TBS (3 times, 10 min), and incubated for 1 h in avidin-biotin complex (ABC in SUMI, Vector Laboratories Inc., Burlingame, CA). Following incubation, slides were rinsed in TBS (3 times, 10 min). The reaction product was visualized by incubation in 1% diaminobenzidine (DAB) (0.05% nickel ammonium sulphate was added to the DAB solution to darken the reaction product) with 0.01% H_2_O_2_ for 7 min. After incubation, slides were rinsed with water. Finally, slides were run through ethanol and xylene and covered for observation by light microscopy.

### Analysis of Fos immunoreactivity

Fos immunoreactivity in the LHA was identified and displayed with a computerized image analysis system consisting of a Zeiss Axioskop and a Media Cybermetrics evolution 9801 video camera (Media Cybernetics, Silver Spring, MD, USA). The LHA was manually outlined in every captured image. The Fos-postive nuclear profiles were manually counted using locally programmed software developed at the Netherlands Institute for Neuroscience. Quantification of Fos was performed by an experimenter who was blind to the experimental conditions. For each rat, one section was measured every 1.80 mm (from bregma −1.20 to −4.56 mm). Subsequently, the mean number of Fos-positive cells in these sections was calculated.

### Statistics

All data are presented as means ± SEM. Statistical analysis was performed using a repeated-measure analysis of variance (rmANOVA) (SPSS Inc, Chicago, USA) to test for effects of *time, stimulation* and time * stimulation interaction. When a treatment or interaction effect was detected, a paired-samples *t*-test test was used to test for group differences. Data were tested on outliers with the Grubbs'outlier test (GraphPad Sofware, Inc, La Jolla, USA). Fos immunoreactivity was statistically analyzed using the non-parametric Kruskall Wallis test. A difference was considered significant when *p* < 0.05 and as a trend when *p* < 0.10.

## Results

### Histology

**Figure 1:**
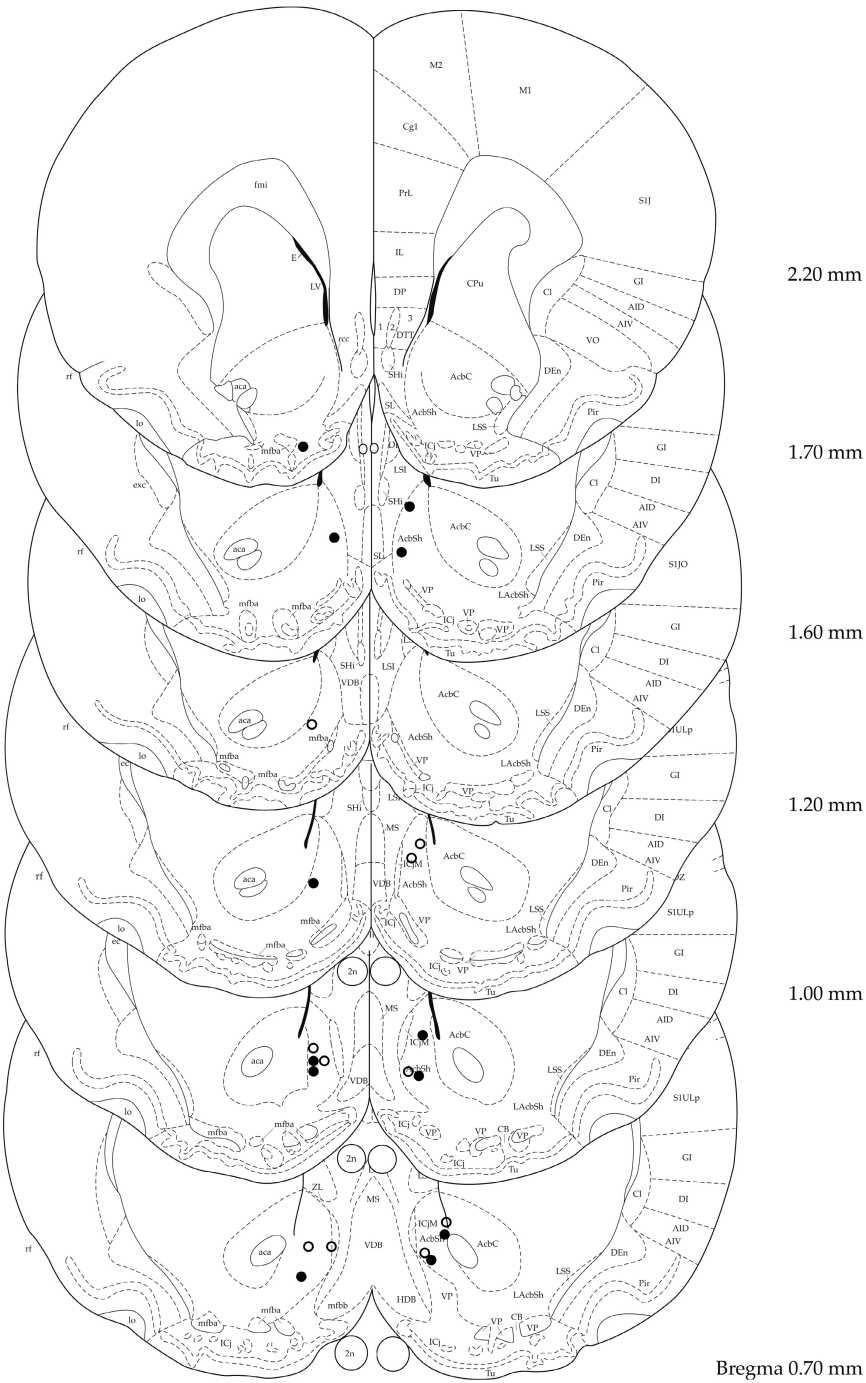
**Localization of electrode tips in 100 μA (black circles) and 200 μA (white circles) stimulated animals**. Adapted from (Paxinos and Watson, 1998).

Figure 1 shows electrode placement of all animals that were bilaterally stimulated in the target area and were included in the analysis. Verification of electrode placement revealed correct placement of the electrodes in the sNAc in 6 out of 12 and 5 out of 13 animals in the 100 and 200 μA groups, respectively. Data of animals with misplaced electrodes were analyzed per condition (100 μ A: *n* = 3, 200 μ A: *n* = 5) and used to assess the topographical specificity of sNAc stimulation. In four animals (*n* = 2 for both stimulation conditions), electrode placement could not be verified due to absence of traces in brain tissue. Two animals could not be used for analysis due to incomplete data sets.

### Concentrations of blood glucose and plasma glucoregulatory hormones

Baseline concentrations of blood glucose and plasma concentrations of glucagon, insulin and corticosterone were not significantly different between the 100 μ A, 200 μA or sham condition (Table 1).

**Table 1:** **Basal concentrations of blood glucose, plasma glucagon, corticosterone and insulin in the 100 (*n* = 6) and 200 μA (*n* = 5) stimulated animals and their sham condition**.

	**sham 100 μ A**	**DBS 100 μ A**	**sham 200 μ A**	**DBS 200 μ A**
Glucose (mmol/l)	6.4 ± 0.1	6.6 ± 0.1	6.6 ± 0.1	6.4 ± 0.1
Glucagon (ng/l)	87.5 ± 5.5	89.9 ± 6.7	77.2 ± 11.7	81.0 ± 8.1
Corticosterone (nmol/l)	33.1 ± 9.5	73.9 ± 35.4	35.0 ± 14.8	44.7 ± 12.2
Insulin (pmol/l) kloppen de	635.9 ± 104.2	692.2 ± 174.0	516.3 ± 135.0	535.9 ± 269.3

Data are means ± SEM.

**Figure 2:**
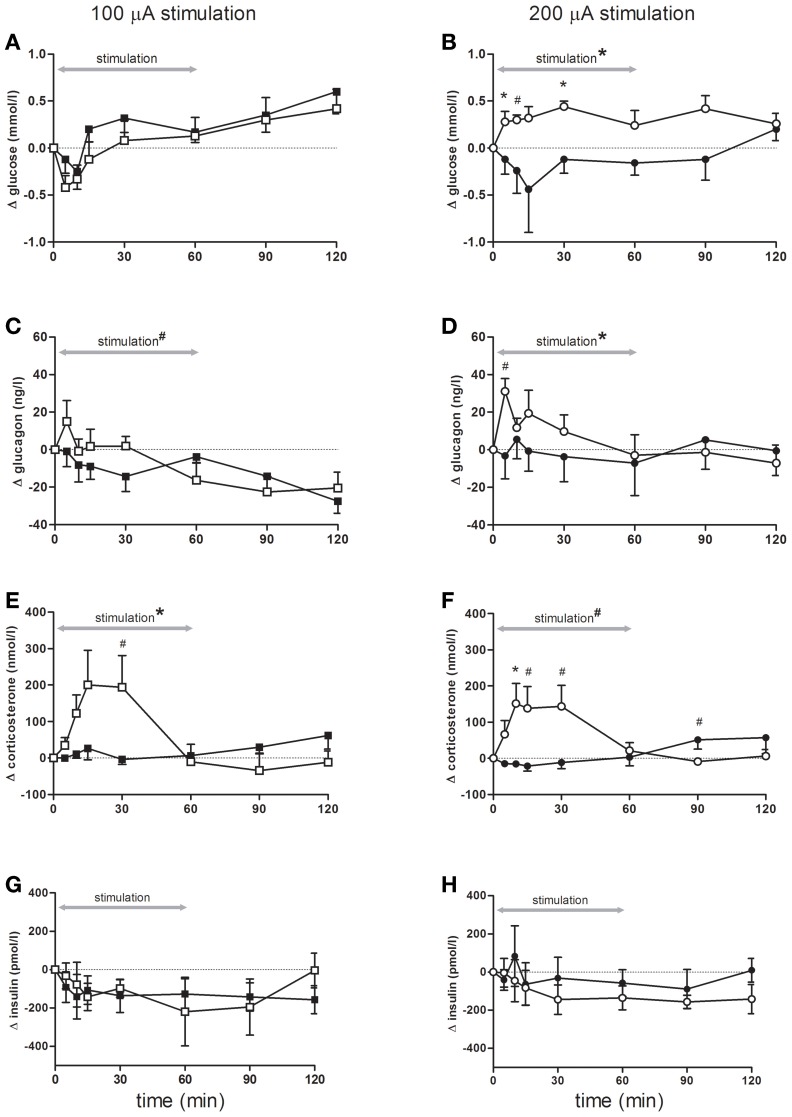
**Blood glucose concentrations (A,B), and plasma- glucagon (C,D), corticosterone (E,F) and insulin (G,H) concentrations during and following stimulation**. Effects of 100 μA stimulation (*n* = 6) are shown in the left column, black squares = sham stimulation, white squares = 100 μA stimulation (**A,C,E,G**), the right column (**B,D,F,H**) shows stimulation at 200 μA (*n* = 5), black circles = sham stimulation, white circles = 200 μA stimulation. All data are presented as mean ± SEM. ^*^p < 0.05, ^#^*p* < 0.10. (**A–B**) Blood glucose concentrations were significantly elevated following stimulation at 200 μA compared with sham stimulation. (A) An overall time effect (*p* < 0.001), but no stimulation or interaction effect. **(B)** rmANOVA indicated an effect of stimulation (*p* = 0.03), post-hoc testing showed that glucose concentrations were significant higher at *t* = 5, and *t* = 30 (both *p* = 0.04), a trend was detected for *t* = 10 (*p* = 0.06). **(C,D)** Plasma glucagon concentrations significantly increased following stimulation at 200 μA compared with sham. **(C)** An effect of time (*p* < 0.001) and a trend for time * stimulation (*p* = 0.09). **(D)** rmANOVA revealed a significant effect of time (*p* = 0.05) and time * stimulation (*p* = 0.03). A trend for higher glucagon elevations was detected at *t* = 5 (*p* = 0.07). **(E,F)** Stimulation at both intensities increased plasma corticosterone concentrations. **(E)** rmANOVA revealed a time (*p* < 0.001) and an interaction effect between time and stimulation (*p* < 0.001). *Post-hoc* analysis revealed a trend for *t* = 30 (*p* = 0.09). **(F)** rmANOVA revealed a trend for time (*p* = 0.10), a significant effect of time * stimulation (*p* < 0.001) and a trend for stimulation (*p* = 0.07). Corticosterone elevation was significant at *t* = 10 (*p* = 0.05) and a trend was detected for *t* = 15, *t* = 30, *t* = 90 (*p* = 0.07, *p* = 0.06 and *p* = 0.08 respectively). **(G,H)** Plasma insulin concentrations were not significant different between the stimulation and sham condition of either 100 or 200 μA stimulated animals.

Although blood glucose concentrations changed over time, there were no differences between rats stimulated with 100 μA compared to controls (Figure 2A). In contrast, blood glucose concentrations showed a significant increase during bilateral sNAc stimulation with 200 μ A, which was significant at *t* = 5 and *t* = 30 compared to the sham condition while a trend was detected for *t* = 10 (Figure 2B, see figure legends for statistics).

Bilateral sNAc stimulation with 200 μA increased plasma glucagon concentrations compared to the sham condition with the highest glucagon concentrations measured at 5 min after DBS onset (Figure 2D). After cessation of stimulation, plasma glucagon concentrations returned to pre-stimulation concentrations and were comparable to plasma concentrations of the sham condition. Statistical analysis showed a significant effect of time, and a trend toward a significant effect of bilateral sNAc stimulation with 100 μA (Figure 2C).

Plasma corticosterone concentrations significantly increased during 100 μA and showed a trend for an increase during 200 μA stimulation when compared to their own sham condition (Figures 2E,F). *Post-hoc* analysis revealed that plasma corticosterone concentrations with 100 μA stimulation showed an increase compared to the sham condition at *t* = 30, although this did not reach significance (Figure 2E).

There were no significant differences in plasma insulin concentrations in animals stimulated with 100 μA or 200 μA compared to their non-stimulation condition (Figures 2G,H).

Statistical analysis of blood glucose and plasma glucoregulatory hormones in the animals with misplaced electrodes revealed a significant increase in plasma corticosterone concentrations during both 100 μA and 200 μA stimulation while concentrations of blood glucose and plasma concentrations of glucagon and insulin were not significantly changed.

### Neural activation in the lateral hypothalamic area

**Figure 3:**
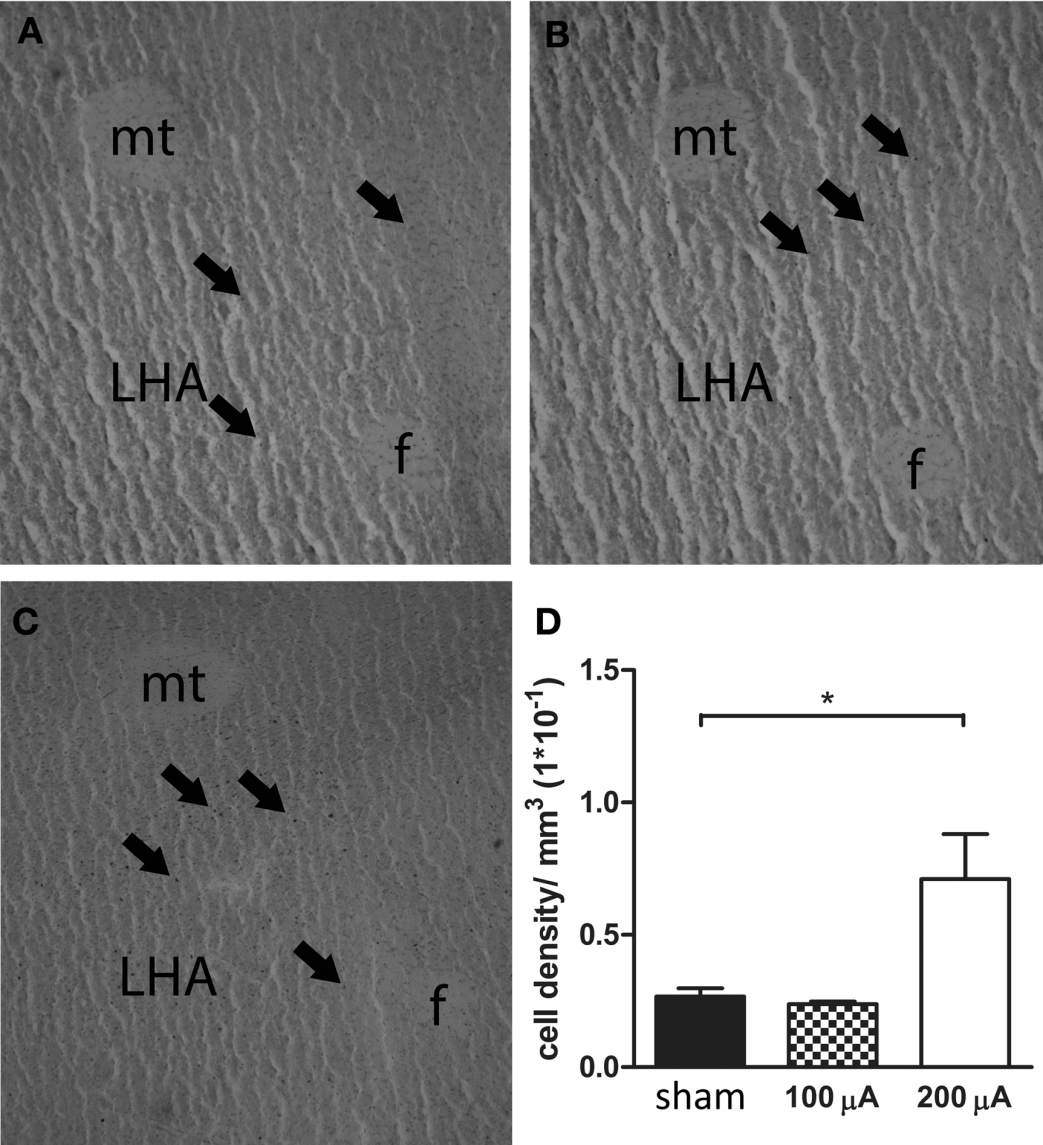
**Representative histological section of a sham (A), 100 μA (B) and 200 μA (C) stimulated rat showing Fos-positive cells in the lateral hypothalamic area (LHA)**. Arrows indicate Fos-positive nuclei. *f*, fornix, *mt*, mammillothalamic tract. **(D)** Number of Fos positive cells in the LHA of sham, 100 μA and 200 μA stimulated animals (*n* = 3–5). Fos staining was significantly increased in 200 μA stimulated animals compared to sham stimulated animals (^*^p = 0.035).

The results of Fos immunoreactivity quantification, and a representative histological section showing Fos-positive cells for each stimulation condition are presented in Figure 3. Quantification of neuronal activity revealed that stimulation of the sNAc with 200 μA increased Fos expression in the LHA compared to no stimulation (sham) or stimulation with 100 μ A. One animal in the 100 μA stimulation group had to be excluded after being identified as an outlier (*p* < 0.05).

## Discussion

We here show that DBS of the sNAc affects systemic concentrations of glucose and glucagon in a region- and intensity- dependent manner. These data thus suggest a role for the sNAc in glucose metabolism through controlling pancreatic and/or hepatic output. Importantly, these data indicate that DBS stimulation used for treating OCD and, which may in future be used for treating eating disorders, may induce metabolic changes.

Following onset of stimulation, concentrations of both blood glucose and plasma glucagon acutely increased and, in the case of glucose, lasted for the duration of the stimulation. These increases were stimulation intensity dependent as DBS at 100 μA did not yield these effects. DBS did not affect plasma insulin concentrations at either intensity tested, but it did increase plasma corticosterone concentrations at both stimulation intensities (100 and 200 μ A). Especially the latter result is of interest as it indicates that DBS of the sNAc can induce a physiological stress-like response, although behavioral experiments in rodents show no increased anxiety or altered locomotor activity after acute sNAc stimulation (van Dijk et al., 2013).

The increase in plasma corticosterone concentrations at both stimulation intensities (100 and 200 μ A), observed in our study, indicates that the higher glucose concentrations are not driven by a DBS-induced increase in general arousal/stress, as only 200 μA increased plasma glucose concentrations. This is further supported by the finding that also in animals with misplaced electrodes plasma corticosterone concentrations increased but blood glucose or plasma glucagon concentrations did not. Importantly, the latter observation suggests that the increased concentrations of glucagon and glucose are specific to DBS of the sNAc as stimulation just outside this area did not evoke a response.

With respect to the neural network that might mediate these effects, it is interesting that the sNAc is anatomically connected to the pancreas, via projections to the LHA and brainstem (Buijs et al., 2001). Indeed, we observed specific activation of the LHA following stimulation, whereas no activation was observed in other areas within the hypothalamus (data not shown). Future experiments are needed to elucidate whether brainstem nuclei are also activated. Interestingly, the Fos expression in the LHA appeared intensity dependent, showing increased expression only at the higher stimulation intensity. This suggests that the higher intensity affects projection areas of the sNAc, whereas the stimulation at 100μA does not, which is also in line with the finding that glucose and glucagon levels were only affected with high intensity stimulation. It is however, unclear at this point which subsets of neurons are activated in the LHA. Several neuropeptides are expressed in the LHA that are known to be involved in energy metabolism. Of special interest are the orexin neurons, which have been shown to receive input from the NAc (Zheng et al., 2003) and are involved in the regulation of glucose metabolism (Yi et al., 2009).

We hypothesize that the increase in blood glucose concentrations is driven by the DBS-stimulated release of glucagon from pancreatic alpha cells. Although the rise in glucagon is small, it has been shown that, in isolated perfused rat livers, small glucagon peaks of 0.4 ug/l induce glycogenolysis and increase glucose concentrations (Sokal et al., 1964). The rise in blood glucose might, however, also result from a direct effect of DBS on the muscle or liver via neural innervation. Sudo et al. (1991), for example, showed that peripheral glucose uptake is under hypothalamic control. Furthermore, we previously showed that the LHA is neurally connected to the liver, and that a GABA antagonist administered to the LHA increased plasma glucose concentrations, which could be prevented by a sympathetic liver denervation (Yi et al., 2009). Administering a GABA antagonist in the LHA did not, however, affect plasma glucagon concentrations making it unlikely that this projection to the liver, if involved, is also underlying the effects of DBS on plasma glucagon concentrations.

The increase in glucagon secretion from pancreatic alpha cells might be achieved via direct stimulation of sympathetic efferents or via sympathetic stimulation of adrenal-norepinephrine (NA) release [eg., Zsombok and Smith (2009); Taborsky and Mundinger (2012)]. As such, it is possible that DBS-induced activation of the HPA-axis contributed to the increase in glucagon via increased NA release. For a more detailed description of these alternative pathways we recommend a recent review by Taborsky and Mundinger (2012). As glucagon secretion is under para- as well as sympathetic control, we cannot distinguish from our data which nervous system is involved. Future experiments could shed light on the relative role of each of these pathways in the regulation of glucose metabolism during DBS and the relative contribution of sympathetic versus parasympathetic activity by combining DBS with the inclusion of independent measures of sympathetic/parasympathetic activity, such as heart rate variation.

In contrast to the effects of DBS on glucagon and glucose, we observed no changes in plasma insulin concentrations, suggesting that DBS of the sNAc does not directly act on pancreatic beta cells. This concept is supported by observations reported by others that electrical stimulation of the ventrolateral hypothalamic area (Helman et al., 1980) and LHA (Helman et al., 1983), induces a rise in glucagon without a rise in insulin. It could be surprising that the glucose increase we observed after DBS did not affect insulin concentrations as *ex vivo* experiments with perfused pancreatic islets from Wistar rats, showed that glucose oscillations with amplitudes between ~0.5 and ~1.5 mmol/L induces insulin secretory oscillations (Chou and Ipp, 1990). However, *in vivo* measurements support our findings by showing that higher glucose oscillations (≥q1 mmol/L) were not accompanied by plasma insulin elevations (Yi et al., 2009). This may suggest that the increase in glucose, observed in animals stimulated with 200 μA, was not sufficient to induce an elevation in plasma insulin concentrations.

To date, the role of the sNAc in the regulation of glucose metabolism has received little attention whereas its role in food-motivated behavior is well established (Diepenbroek et al., 2013). The effects of DBS, we present here, point toward a role for this nucleus in the response to hypoglycaemia. Glucose-sensitive and, to a lesser extent, glucose-receptor cells are present in the sNAc (Papp et al., 2007). In addition, the sNAc is responsive to 2-deoxy-D-glucose (2DG), a glucose analog that inhibits glycolysis (Dodd et al., 2010). Hypoglycaemia could be sensed in the sNAc, and glucose homeostasis would be restored by the secretion of glucagon and stimulation of food consumption. The latter is supported by the study of (Dodd et al., 2010) that showed that the sNAc, as well as the orbitofrontal cortex and ventral pallidum are responsive to 2DG. Together, these regions form a corticostriatal connection with the hypothalamus via which processes of reward can influence the hypothalamic control of feeding behavior (Swanson, 2000; Fulton, 2010) and probably also glucose metabolism.

Apart from showing a functional role of the sNAc in glucose metabolism, these data are of great importance for the clinical use of DBS. These data show that stimulation of the sNAc with DBS for the treatment of psychiatric- and eating disorders may directly affect normal energy homeostasis and induce unwanted side-effects. Although interesting and potentially useful in employing DBS for eating-disorders, these data point out that the effects of DBS are not limited to the brain but also affect peripheral functions which should be taken into consideration when applying DBS.

In summary, we demonstrated that DBS of the sNAc in rats increased blood glucose concentrations and plasma glucagon concentrations in a region and intensity- dependent manner. These data are the first to show a direct relation between the use of DBS in the sNAc and changes in systemic concentrations of glucose and glucagon.

### Conflict of interest statement

The authors declare that the research was conducted in the absence of any commercial or financial relationships that could be construed as a potential conflict of interest.
